# Analysis and Identification of Tumorigenic Targets of MicroRNA in Cancer Cells by Photoreactive Chemical Probes

**DOI:** 10.3390/ijms21041545

**Published:** 2020-02-24

**Authors:** Zhiyu Su, Tsogzolmaa Ganbold, Huricha Baigude

**Affiliations:** School of Chemistry & Chemical Engineering, Inner Mongolia Key Laboratory of Mongolian Medicinal Chemistry, Inner Mongolia University, 235 West College Road, Hohhot, Inner Mongolia 010020, China; suzhiyu@imu.edu.cn (Z.S.); tsogi001@mail.imu.edu.cn (T.G.)

**Keywords:** chemical probe, RNA interference, microRNA target, RNA-seq, tumorigenic gene

## Abstract

Photoactive RNA probes have unique advantages in the identification of microRNA (miR) targets due to their ability for efficient conjugation to the target sequences by covalent crosslinking, providing stable miR-mRNA complexes for further analysis. Here, we report a highly efficient and straightforward method for miR target identification that is based on photo-reactive chemical probes and RNA-seq technology (denotes PCP-Seq). UV reactive probes were prepared by incorporating psoralen in the specific position of the seed sequence of miR. Cancer cells that were transfected with the miR probes were treated with UV, following the isolation of poly(A) RNA and sequencing of the transcriptome. Quantitative analysis of RNA-seq reads and subsequent validation by qPCR, dual luciferase assay as well as western blotting confirmed that PCP-Seq could highly efficiently identify multiple targets of different miRs in the lung cancer cell line, such as targets PTTG1 and PTGR1 of miR-29a and ILF2 of miR-34a. Collectively, our data showed that PCP-Seq is a robust strategy for miR targets identification, and unique in the identification of the targets that escape degradation by miRISC and maintain normal cellular level, although their translation is repressed.

## 1. Introduction

MicroRNAs (miRs) are a group of short (~22 nucleotides) endogenous RNAs that induce RNA interference (RNAi). As one of the key post-transcriptional gene regulators, miRs directly inhibit translational event of a specific group of target mRNAs. Mature miR, upon loading to RNA-induced silencing complex (RISC), binds to a specific region of the target mRNA [1,2]. The results of such binding could result in either (1) an immediate cleavage of the target mRNA by endonuclease (Argonautes) in the miRISC machinery, which results in a swift drop of the cellular level of the target mRNA, and eventually decrease of the corresponding protein level; or (2) a sustained presence of miRISC complex on the target mRNA strand, which is detrimental to the subsequent translation process, leading to the decreased protein content in the cytoplasm, although the target mRNA might retain relative cellular level and eventually decay through deadenylation pathway [3].

As miR mediated post-transcriptional gene regulation plays crucial roles in various biological events in both normal physiological conditions and pathological processes, the identification of the specific targets of a miR is crucial for understanding its biological functions. The seed sequence of a miR, which extends from two to seven nucleotides at the 5′ end, is considered to be the conserved region that recognizes the target mRNA [4]. The heptametrical seed sequence determines that miR can potentially target and downregulate large numbers of target mRNA [3], although the extent of translational repression is different between different targets under physiological and pathological conditions.

miR targets can be predicted by algorithms according to the complementarity-based methods [4,5,6] and thermodynamic-based methods [7,8]. Although miR targets prediction algorithms provide substantial information regarding the miR, the computationally predicted miR targets eventually need experimental validation and, for most miRs, only a small fraction of the predicted targets has been confirmed so far. Target identification that is based on the analysis of crosslinked miR-mRNA complex has shown great potential in the experimental validation of miR target [9,10]. Crosslinking immunoprecipitation (CLIP) based methods [11,12,13,14,15,16,17] capture the partially paired miR-mRNA complex at the target sequence in miRISC through UV crosslinking. Subsequent digestion of protein components in the RNA-protein complex, isolation, and ligation of short RNA fragments, as well as RNA-seq analysis, can accurately identify miR targets. However, multiple steps that were employed in the aforementioned methods may induce artifacts and results in biased conclusions. For example, adaptor ligation of small RNA could be the main contributor to the expression profile bias [18]. Here, we report a synergistic strategy for miR target identification by combining the nucleic acid probe and RNA-seq method (denotes PCP-Seq). This method can potentially identify multiple targets of a given miR simultaneously.

## 2. Results and Discussion

**Figure 1:**
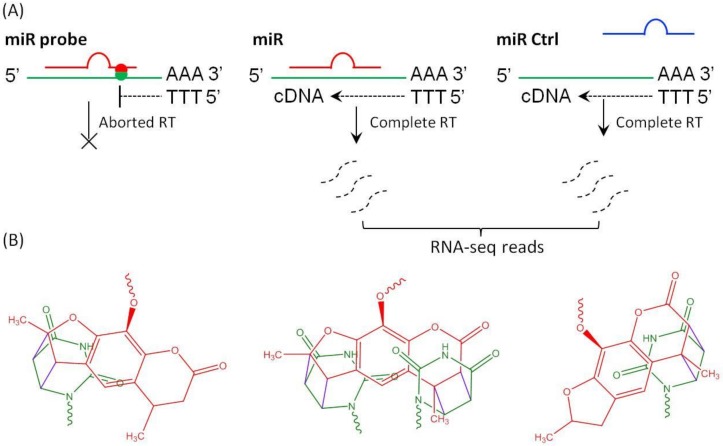
Schematic illustration of photo crosslinking products of the probe and reverse transcription of poly(A) RNA from miR transfected cells. (**A**) Psoralen functionalized miR probe covalently crosslinks to the target mRNA and terminates reverse transcription. RT reaction on miR-mRNA complex is aborted (left), yielding less reads; while control miR (right) or wildtype miR (middle) promotes complete RT reaction and produces equal quantity of RNA-seq reads. (**B**) Photo-addition of psoralen moiety of miR probe to pyrimidine on complimentary sequence of target mRNA produces furan-side monoadduct, pyrine-side monoadduct or diadduct [25].

The rationale for PCP-Seq strategy for miR target identification is as following: upon transfection, psoralen modified miR probe loads to RISC (miRISC), finds and binds to target mRNAs; a subsequent UVA treatment to the live cells produces covalently crosslinked miR probe-target mRNA complex in vivo (*Ago2* knockdown assay previously demonstrated that a psoralen modified miRNA mimic becomes specifically crosslinked to mRNA targets through the RISC complex upon UVA treatment [19], Figure 1), which is then isolated from cell lysate and then subjected to the reverse transcription. As psoralen conjugation to mRNA is a definite stop signal to the reverse transcription (RT) reaction [20,21,22,23,24], the hybrid miR-mRNA does not yield cDNA, which results in decreased reads of target mRNAs in RNA-seq. RT-qPCR, luciferase assay, as well as western blotting could further evaluate the potential candidates from the RNA-seq results. This strategy avoids tedious and excessive manipulations (such as digestion of protein components in RNP, primer ligation, etc.) of miR-mRNA interactome used in the other methods. Consequently, PCP-Seq can effectively reduce biases and provide remarkable advantages over conventional methods, especially for the identification of target mRNAs that resist hydrolysis by miRISC and maintain a normal cellular level, although the translation activity is restricted on those mRNAs.

**Figure 2:**
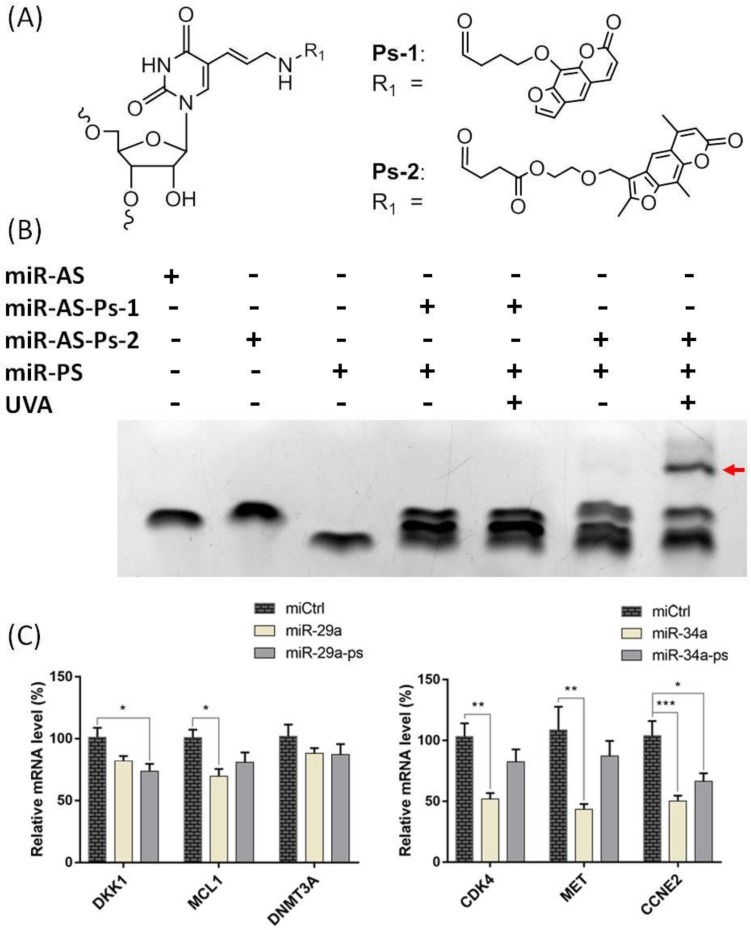
Structures, photo reactivity and bioactivity of psoralen-functionalized miR probes. (**A**) 5-N-U located in the seed sequence of miR reacts with NHS-activated psoralen (Ps-1 and Ps-2) to give photoactive probe (miR-AS-Ps-1 and miR-AS-Ps-2); (**B**) UV reactivity of psoralen modified miR-29a probes was tested for the efficiency of crosslinking between miR-AS-Ps and its complementary strand miR-PS; AS, antisense strand; PS, passenger strand. Red arrow indicates the product of UV mediated reaction; and, (**C**) RNA interference activities of the psoralen-functionalized probes were assessed by RT-qPCR for the representative known targets for miR-29a (left) and miR-34a (right). *ACTB* was served as the internal control. All error bars represent mean ± SD of three independent experiments, and samples with *p* < 0.05 (one-way ANOVA) are marked with asterisks (*, *p* < 0.05; **, *p* < 0.01; ***, *p* < 0.001).

We first prepared miR probes by conjugating psoralen to the seed sequence of miR-29a and miR-34a, according to the previously reported method [19]. miR-29a and miR-34a are both well-known tumor suppressors that are involved in lung cancer or other tumors [26,27,28,29,30,31]. To do this, we used two types of NHS-activated psoralen derivatives (Figure 2A) to conjugate at a specific location in the miRs containing an amine-modified nucleotide (specifically 5-N-U) in the vicinity of the seed sequence. An apparently retarded band for miR-AS-Ps-2 (Figure 2A, lane 2) when compared to miR-AS (Figure 2A, lane 1) confirmed the successful conjugation of psoralen to the miR mimic. A subsequent test for the UVA mediated crosslinking reaction revealed that the miR-AS-Ps-1 probe does not react with its complementary miR-PS strand (Figure 2B), while miR-Ps-2 conjugation showed remarkable UV reactivity and has undergone efficient crosslinking (Figure 2B). This is due to the fact that 4,5′,8-trimethyipsoraien has higher UV reactivity than psoralen under the above-mentioned conditions [32]. We employed miR-AS-Ps-2 for the following experiments based on these observations.

Chemical modification might exert detrimental effects to the functions of biological molecules, including nucleic acids. We first tested the ability of the probes for transcriptional down-regulation of well-established known targets to assess whether or not psoralen modification alters the RNA interference activities of miRs in the cell. To do this, we transfected A549 cells with psoralen functionalized miRs and after 24 h, quantified the mRNA level of established targets, including *DKK1* [33] and *MCL1* [34] for miR-29a, and *CDK4*, *MET*, and *CCNE2* for miR-34a [35] (Figure 2C). The expression level of *DKK1* and *MCL1* was down-regulated by both miR-29a and miR-29a-Ps, although *DNMT3A* [36] (another previously published target of miR-29a) was not affected by both wildtype and psoralen modified miR-29a in A549 cells. Similarly, the mRNA level of *CCNE2* was lowered by both wildtype and modified miR-34a, while *CDK4* and *MET* were also down-regulated by miR-34a-Ps to some extent, which suggested that psoralen modification does not alter the RNA interference pathways, such as miRISC loading, target binding, and subsequent translational inhibitory events.

**Figure 3:**
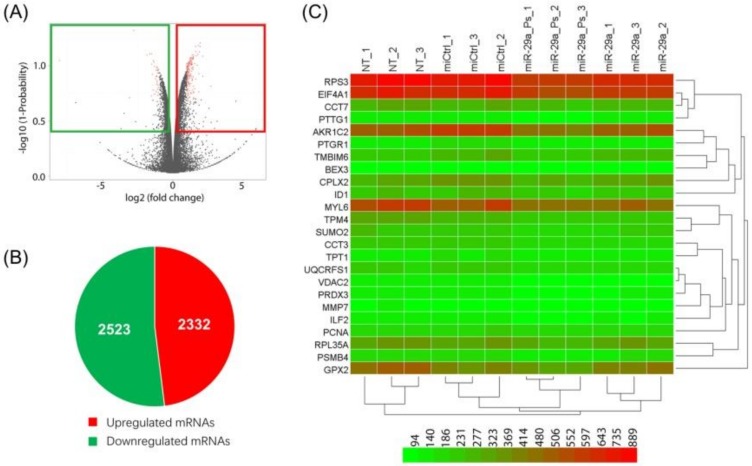
RNA-seq analysis of total RNA isolated from cells transfected with either control miR (miCtrl) or miR probe. (**A**) Volcano plots of mRNAs differentially expressed between miCtrl *vs* miR-29a-ps (left) treated cells; (**B**) Pie charts showing the number of alternatively expressed genes in cells treated with either miCtrl or miR probe; and, (**C**) Differentially expressed genes in miCtrl or miR probe treated cells are analyzed using hierarchical clustering. Each role represents a single mRNA and each column represents one RNA sample. Triplicate samples represent nontreated (NT_1, NT_2, NT_3), miR control (miCtrl_1, miCtrl_2, miCtrl_3), miR probe (miR-x_Ps_1, miR-x_Ps_2, miR-x_Ps_3), and miR (miR-x_1, miR-x_2, miR-x_3). x, 29a.

MiR-29a epigenetically normalizes non-small cell lung cancer (NSCLC) through the suppression of multiple targets. We transfected A549 cells with psoralen functionalized miR-29a probe (denotes miR-29a-ps) and conducted RNA-seq analysis on the poly(A) RNA extracted from transfected cells after *in vivo* UV crosslinking to test the PCP-Seq method and explore potential novel targets of miR-29a. Quantitative analysis of RNA-seq reads (the BioProject accession number, PRJNA559064) revealed that, when compared to the control, a total of 2523 genes were downregulated (log2 (fold change) <−0.2, −log10(*p*-value) > 0.4) in miR-29a-ps treated sample, including the previously identified targets, such as *DNMT3A* (Supporting Information Table S1) (Figure 3). 

**Figure 4:**
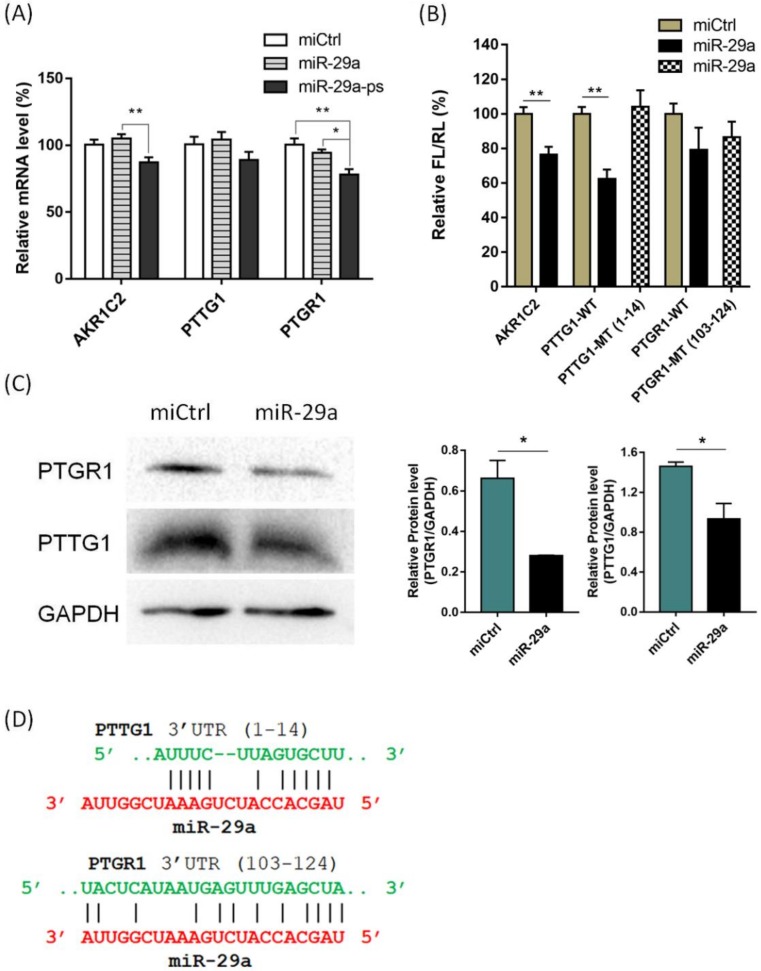
Validation of potential targets of miR-29a. (**A**) qPCR analysis of 3 potential targets that were specifically downregulated only by miR-29-ps; (**B**) Dual luciferase assay showed that *AKR1C2*, *PTTG1* and *PTGR1* have direct binding site of miR-29a in the 3′ UTR region of corresponding mRNA. WT represents wildtype 3′ UTR-containing reporters and MT represents mutant type 3′ UTR-containing reporters; (**C**) Western blotting analysis showed that PTGR1 and PTTG1 protein levels were significantly decreased in the cells transfected with miR-29a; and, (**D**) Potential binding site for miR-29a in the 3′ UTR of *PTTG1* and *PTGR1* mRNA (computed by FIMO [41]). All error bars represent mean ± SD of three independent experiments, and samples with *p* < 0.05 (one-way ANOVA) are marked with asterisks (*, *p* < 0.05; **, *p* < 0.01).

We chose 27 genes with relatively higher reads (Fragments Per Kilobase of transcript per Million, FPKM>100) in nontreated cells to refine the indirect gene regulation and determine the novel direct targets of miR-29a, but showed decreased reads in miR-29a-ps transfected cells according to the RNA-seq results. Quantitative PCR analysis confirmed that 10 out of 27 genes were significantly downregulated (approximately 10 to 30%) by miR-29a-ps (Supporting Information Figure S1, Table S2). Noticeably, several genes (including *AKR1C2*, *PTTG1*, and *PTGR1*) did not change the expression level in the presence of wild type miR-29a, but were considerably downregulated in miR-29a-ps plus UV treated samples (Figure 4A), which suggested that PCP-Seq might be unique for the identification of miR targets that are not cleaved upon miRISC loading.

Next, to further validate the target candidates, the entire 3′ untranslated region (3′ UTR) of 10 potential targets mRNAs were cloned into luciferase-expressing pGL3-Promoter vector, respectively (Supporting Information Figure S3, Tables S3 and S4). HEK293T cells were co-transfected with reporter vectors and wildtype miR-29a. After 48 h, the cells were lysed and luciferase expression was analyzed. The reporters containing 3′ UTR of *AKR1C2*, *PTTG1*, and *PTGR1* showed significantly decreased luciferase expression (approximately 20% to 40%) in miR-29a treated cells, but the vectors harboring mutated miR-29a binding sites did not show significant decrease in luciferase expression, when compared to miCtrl (Figure 4B), confirming that miR-29a has effective binding site(s) in the 3′ UTR of these genes, resulting in the translation repression of luciferase. Next, we investigated the change of protein level of three candidate target genes by using western blotting assay on the total protein obtained from A549 cells transfected with miR-29a. A significantly decreased expression of PTTG1 and PTGR1 was observed, confirming that both are direct targets of miR-29a in A549 cells (Figure 4C). The protein level of AKR1C2 did not decrease in miR-29a transfected cells, which indicated that it might be a false positive (Supporting Information Figures S4 and S5). Furthermore, close examination of the 3′ UTR of *PTTG1* and *PTGR1* elucidates the existence of a possible binding site of miR-29a (Figure 4D). Note that the mRNA level of these two genes did not change upon the transfection of wildtype miR-29a in A549 cells, indicating that miRISC does not immediately hydrolysis the mRNA. Instead, miR-29a inhibits the translational process, resulting in the decreased protein level in the cell. PTTG1 promotes the invasion and migration of human NSCLC cells [37], which is modulated by miR-186. Similar to miR-29a, miR-186 does not alter the mRNA level of PTTG1, but inhibits the protein synthesis [38]. A recent study found that PTGR1 also plays a positive regulatory role in cancer cell proliferation [39,40].

**Figure 5:**
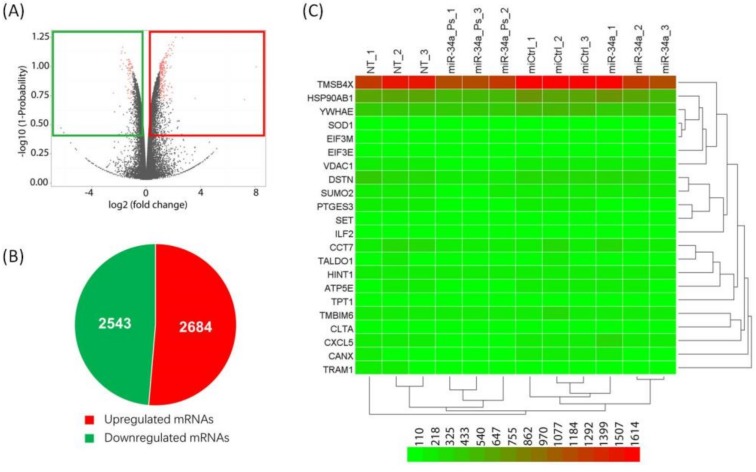
RNA-seq analysis of total RNA isolated from cells transfected with either control miR (miCtrl) or miR probe. (**A**) Volcano plots of mRNAs differentially expressed between miCtrl *vs* miR-34a-ps (left) treated cells; (**B**) Pie charts showing the number of alternatively expressed genes in cells treated with either miCtrl or miR probe; and, (**C**) Differentially expressed genes in miCtrl or miR probe treated cells are analyzed using hierarchical clustering. Each role represents a single mRNA and each column represents one RNA sample. The triplicate samples represent nontreated (NT_1, NT_2, NT_3), miR control (miCtrl1_, miCtrl_2, miCtrl_3), miR probe (miR-x_Ps_1, miR-x_Ps_2, miR-x_Ps_4), and miR (miR-x_1, miR-x_2, miR-x_3). x, 34a.

We prepared psoralen modified probe for miR-34a, a key suppressor of tumorigenesis in various tumor, to test the efficiency of PCP-Seq method on other miRs [42]. A previous study revealed that miR-34a suppresses tumor immune evasion by targeting PDL1 in NSCLC [43]. We transfected psoralen modified probe (miR-34a-ps) into A549 cells. After 24 h, the cells were exposed on UVA (360 nm) radiation, and poly(A) RNAs were extracted. Following a RT reaction using the oligo(dT) primer, the cDNA was subjected to sequencing analysis (the BioProject accession number, PRJNA559064), which gave a total of 2543 downregulated genes (Figure 5).

**Figure 6:**
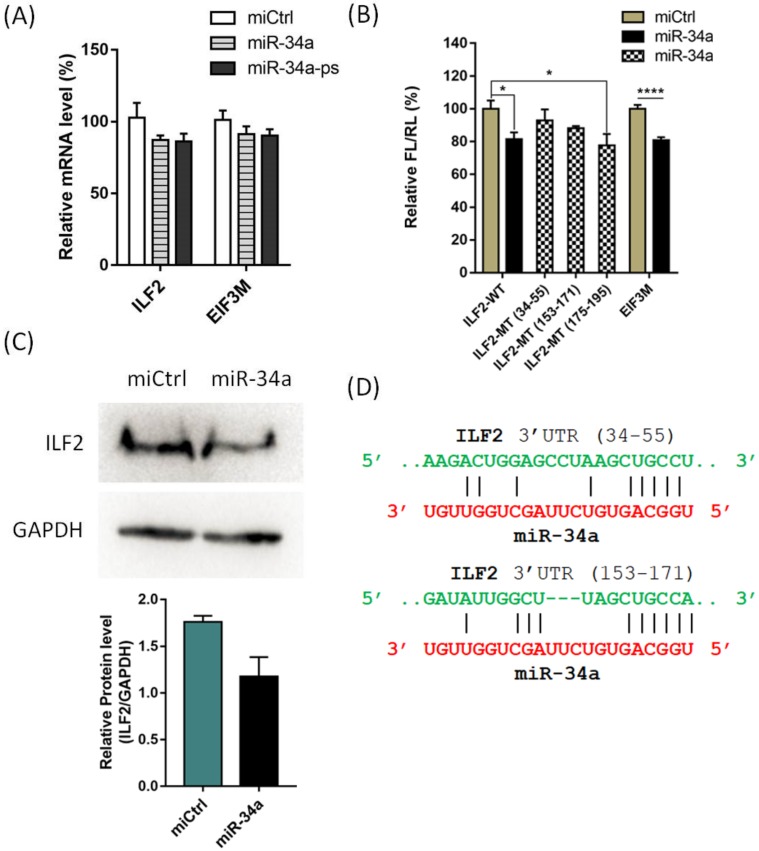
Validation of potential targets of miR-34a. (**A**) qPCR analysis of 2 potential targets that were specifically downregulated by miR-34-ps; (**B**) Dual luciferase assay showed that *ILF2* and *EIF3M* have direct binding site(s) of miR-34a in the 3′ UTR region of mRNA. WT represents wildtype 3′ UTR-containing vectors and MT represents mutant type 3′ UTR-containing vectors; (**C**) Western blotting analysis showed that ILF2 protein level was significantly decreased in A549 cells transfected with miR-34a; (**D**) Potential binding sites of miR-34a in the 3′ UTR of *ILF2* mRNA (by FIMO [41] program). All of the error bars represent mean ± SD of three independent experiments, and samples with *p* < 0.05 (one-way ANOVA) are marked with asterisks (*, *p* < 0.05; ****, *p* < 0.0001).

A subsequent qPCR validation of the RNA-seq result confirmed that the expression of 10 out of 34 genes were downregulated by miR-34a-ps (approximately 10 to 20%) when compared to the control (Supporting Information Figure S2, Table S2). Next, 3′ UTR of these 10 abundantly expressing candidate genes were cloned into downstream of luciferase coding reporter vectors, respectively. The HEK293T cells were co-transfected with respective plasmids and wildtype miR-34a or miCtrl, and the luciferase expression was detected while using the dual luciferase assay after 48 h. The expression of luciferase in two constructs containing the 3′ UTR of interleukin enhancer binding factor 2 (*ILF2*) and Eukaryotic translation initiation factor 3 subunit M (*EIF3M*), respectively, was significantly decreased, which suggested that *ILF2* and *EIF3M* mRNA have direct binding site of miR-34a in the 3′ UTR (Figure 6A,B). Further analysis by western blotting showed that ILF2 protein level dramatically decreased in A549 cells 48 h after the transfection of miR-34a as compared to the miCtrl (Figure 6C,D), thus confirming that ILF2 is a direct target of miR-34a. We were not able to further validate EIF3M due to the unavailability of antibody. An oncogene, *ILF2* promotes tumorigenesis in several cancers, including NSCLC. A previous study has discovered that *ILF2* is a direct target of miR-7 in pancreatic cancer cells [44]. To the best of our knowledge, this report is the first to identify *ILF2* as a direct target of miR-34a in the NSCLC cell line.

## 3. Materials and Methods

### 3.1. Preparation of Psoralen Modified miRNA Probes

The chemical probes were prepared according to the previous report [19]. Briefly, 2′-ACE protected, 5-N-U modified antisense (sense strand) microRNA mimic was dissolved in DMSO. A DMSO solution of SPB (succinimidyl-(4-(psoralen-8-yloxy))-butyrate, NHS-Psoralen; Thermo Fisher Scientific, Waltham, MA, USA), and triethylamine were added (molar ratio of microRNA: SPB, 1:50). The reaction mixture was incubated at room temperature for 24 h. Subsequently, ethyl acetate was added, and the precipitate was collected by centrifugation. The pellet was resuspended in ethyl acetate and washed repeatedly. After air-drying the pellet, 2′-deprotection buffer was added, following heating at 60 °C for 2 h to give miR-AS-Ps-1. The miR probe containing 4,5′,8-trimethylpsoralen (TMP) was synthesized according to the previous report [19]. Briefly, 4′-(2-hydroxyethoxy)methyl-4,5′,8-trimethylpsoralen was reacted with succinic anhydride in THF. Afterwards, *N*-hydroxysuccinimide (NHS) and *N*-(3-(dimethylamino)propyl)-*N*′-ethylcarbodiimide hydrochlorides (EDC) were added to the reaction mixture. The final product was purified using a silica gel chromatography. The NHS-activated TMP was conjugated to miR in a similar way described previously to give miR-AS-Ps-2. The purity of miR-AS-Ps-1 and miR-AS-Ps-2 was monitored by denaturing polyacrylamide gel electrophoresis. For annealing, an equimolar amount of deprotected antisense (guide) strand and the passenger strand were mixed, followed by heating at 60 °C for 10 min. then incubated at room temperature for 30 min. The sequence of 5-N-U modified microRNA mimics that were used for preparation of the probes were as following: hsa-miRNA-29a: antisense strand: 5′-UAGCACCA(5-N-U)CUGAAAUCGGUUA-3′, sense strand: 5′-UAACCGAUUUCAGAUGGUGCUA-3′. hsa-miRNA-34a: antisense strand: 5′-UGGCAGUG(5-N-U)CUUAGCUGGUUGU-3′, sense strand: 5′-ACAACCAGCUAAGACACUGCCA-3′.

### 3.2. MiR Probe-Mediated Crosslinking and RNA-Seq Analysis

The A549 cells were seeded in six-well plate (5 × 10^5^ cells per well), maintained in Dulbecco’s Modified Eagle Medium: Nutrient Mixture F-12 (DMEM/F12) (Biological Industries, USA) that was supplemented with 10% (*v*/*v*) fetal bovine serum (FBS) (Biological Industries, USA) and 1% (*v*/*v*) penicillin/streptomycin mixture (Gibco, Waltham, MA, USA). After the cell confluency reached about 70%, psoralen modified double stranded miR probe, or wildtype miR, or nontargeting control (miCtrl) was transfected at 50 nM final concentration with lipofectamine 2000 (Invitrogen, Carlsbad, CA, USA), respectively. After 24 h, the cells were treated with UVA radiation (360 nm) in 1 × PBS for 10 min. at room temperature. Then, total RNAs were extracted with TRIzol reagent (Thermo Scientific, Waltham, MA, USA). Afterwards, the isolation of poly(A) RNA, reverse transcription was conducted while using oligo(dT) primer and the obtained cDNA was subjected to sequencing (BGISEQ-500 RNA-Seq analysis, BGI).

### 3.3. Dual Luciferase Assay

The 3′ UTRs of candidate mRNAs were cloned by using In-Fusion cloning assay with In-Fusion HD Cloning Kit (Takara Bio, Dalian, China) into the *Xba* Ι site of firefly luciferase-expressing pGL3-Promoter vector, respectively. Corresponding mutant type reporters were constructed by using reverse PCR assay (the constructed vectors were used for templates and the mutant-containing sequences were used for primers), and then linear amplification products were cyclized by homologous recombination with In-Fusion HD Cloning Kit. The HEK293T cells were seeded in 48-well plate (5 × 10^4^ cells per well), maintained in DMEM/F12 (Biological Industries, USA) that was supplemented with 10% (*v*/*v*) fetal bovine serum (Biological Industries, USA) and 1% (*v*/*v*) penicillin/streptomycin mixture (Gibco). After 24 h, 350 ng of constructed either wildtype or mutant type vector and 35 ng of pRL-TK control vector (renilla luciferase-expressing) were co-transfected with either miCtrl or miR at 50 nM final concentration while using lipofectamine 2000 (Invitrogen). The cell lysates were harvested after 48 h, and firefly/renilla luciferase (FL/RL) ratios were detected with Dual-Glo Luciferase Assay System (Promega, Madison, WI, USA), following the manufacturer’s protocol.

### 3.4. Western Blotting Assay

The A549 cells were seeded in six-well plate (5 × 10^5^ per well). After 24 h, either miCtrl or miR (final concentration at 50 nM) were transfected with lipofectamine 2000, respectively. Two days later, the cell lysates were harvested and the total protein was extracted with M-PER Mammalian Protein Extraction Reagent (Thermo Fisher Scientific, Waltham, MA, USA). The concentration of total proteins was measured with Pierce BCA Protein Assay Kit (Thermo Fisher Scientific, Waltham, MA, USA). Equal amounts (30 µg total proteins), either from miCtrl or miR treated cells, were loaded and separated on SDS-PAGE gel electrophoresis. The proteins were transferred to membrane and incubated with antibodies (Anti-ILF2 antibody, 1:3000, ab154791; anti-Securin antibody, 1:5000, ab79546; anti-PTGR1 antibody, 1:2000, ab181131; anti-AKR1C1/AKR1C2 antibody, 1:3000, ab179448; anti-PCNA antibody, 1:3000, ab92552; anti-Peroxiredoxin 3 antibody, 1:10000, ab128953; anti-GAPDH antibody, 1:10000, ab128915; Abcam, Cambridge, MA, USA). After incubation with secondary antibody (Goat Anti-Rabbit IgG H&L (HRP), 1:4000, ab97051, Abcam), the images were acquired by using chemiluminesence detection.

## 4. Conclusions

When compared to tedious and time-consuming CLIP-based methods, PCP-Seq avoids complicated protocols, such as recombination of overexpressed vectors, enzyme digestion, RNA linker ligation, protein purification, and so on, and identifies miR targets conveniently and efficiently (Table 1). All PCP-Seq needs is to design a unique miR mimic modified with photo-reactive chemical group (psoralen).

Several databases [45,46,47] are now available for the reference of miR targets that has been computationally predicted. However, the number of confirmed targets of specific miR is far from the coverage of the whole targetome. The identification of novel targets of miR depends largely on straightforward strategy and robust methodology. The synergistic PCP-Seq strategy successfully identified, validated, and confirmed multiple novel targets of two distinct miRs in the cancer cell line. PCP-Seq might provide advantages, not only for the identification of target mRNAs, but also for monitoring the translation activity of the target mRNAs. Moreover, PCP-Seq might also be applied for the identification and monitoring of noncoding RNAs, such as long noncoding RNAs (lncRNAs). PCP-Seq is also a candidate method for either the diagnosis and treatment of genetic disorder induced diseases, such as cancers, Alzheimer’s disease, Duchenne muscular dystrophy (DMD), and so on, uncovering the physiological processes and biological functions of those RNAs. Collectively, our data demonstrated that the novel strategy of PCP-Seq is efficient, straightforward, and can be applied to any miRs to identify multiple targets in any cancer cell lines.

## Figures and Tables

**Table 1:** Comparison of methodologies used in PCP-Seq strategy with previous CLIP-based methods.

Protocol	PCP-Seq	CLIP [11,12]	HITS-CLIP [13]	PAR-CLIP [14,15]	CLASH [16,17]
UV radiation	360 nm	254 nm	254 nm	360 nm	254 nm
Photoactivatable nucleosides	✓	✕	✕	✓	✕
Total RNA isolation	✓	✕	✕	✕	✕
Partial RNase digestion	✕	✓	✓	✓	✓
Creation of recombinant protein	✕	✕	✕	✕	✓
Generation of stable cell line	✕	✕	✕	✕	✓
Recombinant protein purification	✕	✕	✕	✕	✓
Immunoprecipitation	✕	✓	✓	✓	✕
Phosphatase treatment	✕	✓	✓	✓	✓
3′ or 5′ RNA linker ligation	✕	✓	✓	✓	✓
Radiolabeling of RNA segment	✕	✓	✓	✓	✓
Purification on SDS-PAGE	✕	✓	✓	✓	✓
RNA purification	✕	✓	✓	✓	✓
Reverse transcription	✓	✓	✓	✓	✓
PCR amplification	✓	✓	✓	✓	✓
High-throughput sequencing	✓	✕	✓	✓	✓

PCP-Seq—Photo-reactive Chemical Probes and RNA-seq; CLIP—Cross-linking and Immunoprecipitation; HITS-CLIP—High-throughput Sequencing of RNAs Isolated by Crosslinking Immunoprecipitation; PAR-CLIP—Photoactivatable-Ribonucleoside-Enhanced Crosslinking and Immunoprecipitation; CLASH—Cross-linking, Ligation, and Sequencing of Hybrids.

## Supplementary Materials

Supplementary materials can be found at https://www.mdpi.com/1422-0067/21/4/1545/s1. Figure S1: qPCR validation of RNA-Seq result (27 genes); Figure S2: qPCR validation of RNA-Seq result (34 genes); Figure S3: Cloning of 3′ UTR of candidate targets genes and construction of luciferase expressing vectors; Figure S4: Western blotting analysis showed that the protein level of AKR1C2 did not decrease in miR-29a transfected cells, indicating that it may be a false positive; Figure S5: Validation of potential targets (*PRDX3*, *PCNA* and *BEX3*) of miR-29a; Table S1: FPKM value of established targets in RNA-Seq; Table S2: List of primers used for qRT-PCR; Table S3: List of primers used for constructing reporter vectors; Table S4: List of primers used for reverse PCR.
